# Assessment of the Spatial Distribution of Moisture Content in Granular Material Using Electrical Impedance Tomography †

**DOI:** 10.3390/s19122807

**Published:** 2019-06-23

**Authors:** Jan Porzuczek

**Affiliations:** Faculty of Environmental Engineering, Cracow University of Technology, Warszawska 24, 31-155 Krakow, Poland; jan.porzuczek@pk.edu.pl

**Keywords:** drying process, Electrical Impedance Tomography, impedance spectroscopy, chokeberry, EIDORS

## Abstract

This paper presents a method for the online determination of the spatial distribution of the moisture content in granular material. It might be essential for the monitoring and optimal control of, for example, drying processes. The proposed method utilizes Electrical Impedance Tomography (EIT). As an exemplary material for experimental research, the black chokeberry (Aronia melanocarpa) was used. The relationship between the electrical impedance of the chokeberry and its moisture content was determined for a wide range of frequencies (20 Hz–200 kHz). The EIT research consisted of both simulation and experimental investigation. Experimental studies of the spatial distribution of the moisture content were performed in a cylindrical vessel equipped with 8 electrodes circumferentially arranged. The voltage signal from the electrodes was acquired simultaneously using the data acquisition module. Due to the high impedance of the chokeberries, exceeding 10^9^ Ω for the dried matter, extraordinary instrumentation was necessary to be applied. On the other hand, raw chokeberry was characterized by a several orders of magnitude lower impedance (10^3^–10^4^ Ω), especially for high frequencies. The wide range of the observed impedance was able to be measured owing to its use of the voltage stimulation instead of the current stimulation (which is most common for EIT). The image reconstruction problem was solved using an iterative Gauss–Newton algorithm and the EIDORS (Electrical Impedance Tomography and Diffuse Optical Tomography Reconstruction Software) package. The obtained results showed a satisfactory ability to localize an insufficiently dried part of the material. Prospective ways to improve the imaging quality are also discussed.

## 1. Introduction

Measurement of moisture content in granular or solid materials is vital for a number of technological processes (e.g., drying or storage in silo). This measurement might be also be an essential tool for the monitoring the states of buildings or other works of construction. The inspection of dampness in building foundations and the leakage of flood embankments are possible examples. This paper presents research focused on the assessment of the spatial distribution of moisture content in granular material during its drying. However, the results acquired in this study might also be adapted to other applications that require similar measurements.

Drying processes (e.g., agricultural crops processing or fertilizers production) are known to be one of the most energy consuming processes. Therefore, any improvement in such processes may lead to significantly reduced energy consumption and, thus, ecologic as well as economic benefits. One of possibilities for optimizing the drying process is a proper process control strategy taking advantage of modern measurement techniques and instrumentation. The main purpose of this paper is to study the prospect of applying Electrical Impedance Tomography (EIT) is a non-intrusive measurement technique that could allow the drying of granular materials to be monitored online. The use of such a method would give better control to a process and may contribute in the improvement of the product’s quality as well as reducing its energy consumption. As an exemplary material for experimental research, the black chokeberry (Aronia melanocarpa) [1] was used, but it appears possible to use this technique for a range of other agricultural crops (e.g., currants and cranberries) or other conductive granular materials (e.g., fertilizers and biomass).

The term ‘tomography’ is common for all measurement and visualization methods that provide information about the spatial distribution of certain material properties within an examined object, using measurements performed at the border of the object [2,3]. The graphical representation of those properties’ distributions can be achieved as a result of measurement. Originally, this technique was used in medicine, but since the early 1990s, the first engineering applications of tomography appeared, leading to the development of the modern measurement technique known as ‘process tomography’. The interest in the utilization of electrical phenomena in tomography contributed to the origin of electrical tomography, which was relatively inexpensive in application. A main advantage of all the tomographic methods may also include non-intrusiveness. Depending on the electrical and magnetic properties of the examined object, different measurement methods can be applied [4,5]:

- Electrical Impedance Tomography (EIT): The distribution of electrical conductivity is determined within the examined object.
- Electrical Capacitance Tomography (ECT): ECT allows one to determine the permittivity distribution in an area filled with dielectrics.
- Electromagnetic Tomography (EMT) or Magnetic Induction Tomography (MIT). These techniques are used to determine the permeability distribution in the examined object.

Application of tomography for the assessment of moisture content in the material-of-interest is not a new idea. However, the most common applications utilize ionizing radiation tomography [6] or ultrasound tomography [7]. In the literature, one can find a few examples of the use of electrical tomographic methods for monitoring of the drying processes [8,9], but they relate mainly to applications in pharmacy and use capacitance tomography instead of EIT, as proposed in this paper. Application of the Electrical Capacitance Tomography is suitable in the case of highly dielectric materials or processes in which the contact between electrodes and materials is inadvisable (e.g., fluidized bed processes, due to electrode erosion) [3,10]. Impedance tomography seems to be more adequate than ECT for the considered purpose. This is due to the relatively high conductivity of the wet material, which is typical for plant materials and many chemical substances [11], as well as a meaningful relationship between a material’s conductivity and its moisture content. This feature was already implemented in research on building wall dampness [12] and the monitoring of waste landfills [13]. So far, no studies have been carried out to determine the moisture content of granular materials using EIT. On the other hand, in the state of low moisture content, a significant reduction of material conductivity might be expected. This results in the increased complexity of measurement appliance. In particular, utilizing the current stimulation commonly used in EIT is, in this case, virtually impossible. Instead of this, a voltage source should be used. The high impedance of the dry material shows the need to accurately measure very low current (a few μA). Particular attention must be given to ensuring the best possible contact between the material and the sensor electrodes. For future research, the use of multifrequency impedance tomography or spectro-tomography [14,15,16] seems to be promising.

The program of the research presented in this work consisted of three stages. In the first stage, the dependence between the complex impedance and the moisture content of the tested material was experimentally determined for a wide frequency range. In a later part of the research, a series of computer simulations were carried out. The aim of these simulation was analysing the imaging quality obtainable for the given measurement system and the selected algorithm for image reconstruction (Gauss-Newton algorithm [2]). The last part of the research was the experimental verification of the ability to identify the material with increased humidity in the sensor area. Experimental research was carried out for three frequency values of the stimulating voltage.

The experimental investigation proved the significant dependence between the absolute impedance of the chokeberry and its moisture content. This dependence showed a good potential to use it for an EIT purpose. The simulation analysis showed that even if some limitations might be expected, the 8-electrode EIT system with larger electrodes can provide a rewarding imaging quality, especially for process control purposes. The validation of the imaging quality of the spatial distribution of moisture content using the 8-electrode EIT system, conducted experimentally, confirmed the simulation results. The artefacts that appeared in the reconstructed images tended to decrease with a raise in frequency.

## 2. Materials and Methods

### 2.1. Impedance Spectrum of the Chokeberry and Its Dependence on Humidity—The Method of Examination

Impedance spectroscopy is the research method that allows one to obtain the complex impedance of the investigated system as a function of frequency [11]. After the insertion of the conductive material between two electrodes, such a system can be regarded as a combination of several passive elements, such as resistors and capacitors. Depending on the adopted structure, the model will approximate the experimental results with a certain level of accuracy. For the material originating from plants, the first model, consisting of three elements, was proposed by Hayden at the turn of the 1960s and 1970s [17]. This model was then widely used by other researchers [18]. However, examples utilizing more sophisticated models can be found. For instance, in [19], the authors proposed a model consisting of five elements. According to the authors, this model better represents the electrical properties of the plant cells. The model proposed by Zhang was subsequently used during the impedance spectroscopic investigation of kiwi fruit [20]. In the present paper, the simplification of the Hayden model was used. This system is modelled as a resistor and capacitor connected in parallel. The reason for such a simplification is the relatively narrow frequency range in comparison to the above-mentioned research.

As mentioned, after the insertion of the conductive material (in this case, chokeberry) between two electrodes, such a system can be regarded (as a simplification) as a parallel combination of a resistor *R* and a capacitor *C* [11]. The reactance *X* of the capacitor shows the dependence of angular frequency *ω* as shown in Equation (1):

(1) $$X=\frac{1}{\omega C},$$

Likewise, complex impedance of the circuit, Equation (2), is a function of angular frequency ω:

(2) $$Z=\frac{RX^{2}}{R^{2}+X^{2}}+j\frac{R^{2}X}{R^{2}+X^{2}},$$

Complex impedance can be equivalently represented as in Equation (3), using the absolute impedance |Z| and the phase angle *ϕ*:

(3) $$Z=\left|Z\right|\cdot e^{-j\phi },$$

For a given measurement system, both the absolute impedance |Z| and the phase angle *ϕ* are related to the condition of a material being investigated (temperature, humidity) and that relationship might be obtained experimentally. On the other hand, knowledge about that relationship leads to the conclusion of the state of the material by measuring the above-mentioned values.

According to the literature data [1], initial humidity of fresh harvested chokeberry is typically 71.2%–84.8%. The chokeberry frozen immediately after harvest was used for the experimental research. The material contains whole fruits, without any mechanical processing and damages This is noteworthy, since in the case of processed chokeberry (e.g., chopped or pressed), a peel of the fruit gets damaged. Thus, in such a situation, the results described below cannot be reliable [21]. After defrosting and reaching room temperature, a thin layer of water on the surface of the peel still existed. This state of berries was assumed to be the initial state. In a further step, the chokeberry was dried in the open air at a temperature of 20 °C and relative air humidity of 35%–40%. The sample of material (approximately 0.75 dm^3^) was gathered periodically and the measurement of absolute impedance and phase angle was taken using the procedure described below. Using the moisture analyser Radwag MAC 210, the humidity of the chokeberry sample was designated afterward. A measurement was taken for the chokeberry’s humidity range of 80.0%–12.6%. In the case of low water content (<20%), the impedance exceeded the measuring range of the instrument reaching more than 10^9^ Ω (for low frequency).

The study was conducted in the measurement vessel, as detailed in Section 2.3, at a fixed temperature of 20 °C. For the purpose of verification, the procedure was repeated several times for each sample of the chokeberry. This repetition involved both changing the measuring electrodes and removing and re-inserting the material into the area of the sensor. Each time the observed differences did not exceeded a few percent, and this is most likely the result of differences in the contact impedance between the electrode and the berries, as well as between the berries themselves at different arrangements of the material. 

The impedance of the chokeberry was measured using a programmable LCR bridge Rhode-Schwarz Hameg HM 8118. This device allows one to designate selected parameters of the circuit (in this case absolute impedance and phase angle) for 69 frequency values in the range of 20 Hz–200 kHz. The frequency range as well as the number of measurement points were limited by the LCR bridge specification. Since the LCR bridge is equipped with both RS232 and USB communication interfaces, allowing one to control the device using a computer, it was possible to automate the measurement procedure for the entire frequency range.

### 2.2. Simulation Analysis of Projected EIT Image Quality

Determination of the conductivity distribution in the cross-section of a sensor, based on the measured voltage, requires a two-step process. The aim of the first stage is to solve the forward problem, leading to a designation of the transformation matrix of the measurement system. This step is based on the Laplace Equation (4), which defines the distribution of electrical potential *V* in the studied area with the material of conductivity *σ* [2]:

(4) ∇·σ∇V=0,

The solution of Equation (4) for the given measurement system is usually carried out with the Finite Elements Method. Introducing *v* as the vector of measured potentials at the border of the sensor leads to the designation of the transformation matrix **T**, satisfying the Equation (5):

(5) v=T⋅σ,

Reconstruction of the conductivity image is the second stage of the analysis of acquired data. The image is constructed by solving the inverse problem defined by Equation (6).

(6) $$\sigma =T^{-1}\cdot v$$

However, the T matrix is not a square one, so it is not possible to use conventional methods for determining the inverse matrix. This problem has resulted in a number of algorithms for image reconstruction, approximating the solution of the inverse problem. Starting from linear methods (e.g., Linear Back-Projection) through nonlinear and iterative methods (e.g., Gauss–Newton) [2,3,5], the approach to the inverse problem has evolved into a dozen modern algorithms that utilize so-called artificial intelligence methods. For instance, in [22], the authors reviewed almost 70 papers describing novel methods for image reconstruction. Some of the modern, advanced algorithms, e.g., Sparse Bayesian Learning [23], are thought to offer a better resolution for imaging in comparison to classical methods.

Due to the ill-conditioning of the inverse problem, the solution obtained may not correctly reflect the distribution of the analysed properties in the area of the sensor. The feasibility of the correct imaging and the projected image quality should by verified using simulation studies. For this reason, the experimental studies were preceded by conducting a series of simulation analyses that gave an overview of the predicted quality of imaging in a considered EIT system. The use of specialized software, for example EIDORS (Electrical Impedance Tomography and Diffuse Optical Tomography Reconstruction Software) [24,25], allows both the simulation test of impedance tomography system and the reconstruction of images based on experimental data. Reconstruction of the image in the software package is possible using a number of algorithms. A broad overview of the application of the EIDORS software was presented by Holder [2]. Commercially available EIT systems usually come with proprietary software, e.g., the ITS Reconstruction Toolsuite [26], which may be used alternatively.

The simulation research consisted of two main stages. In the first stage, the investigation was focused on the possibility of identifying the region that is characterized by a different moisture content than the rest of the material. In the scope of this research, the impact of the diameter of the area with increased humidity, as well as its position inside the sensor area, on detection capability was investigated. In this study, the circular EIT sensor with an internal diameter of 11 cm was used. A simulation study was conducted for the situation in which an area of higher humidity exists inside the sensor filled with the chokeberry. That area had a circular shape with a diameter 60 mm or 30 mm, located in the centre of the sensor or near its border. Four cases were analysed:

- Area of diameter 30 mm located near the border of the sensor;
- Area of diameter 60 mm located near the border of the sensor;
- Area of diameter 30 mm located in the centre of the sensor;
- Area of diameter 60 mm located in the centre of the sensor.

The rest of the material had homogeneous moisture content. Using the EIDORS software together with Netgen meshing software [27], the model of the measurement vessel, electrodes, and distribution of material inside the vessel was created, and a finite element grid was generated. Each simulation was performed both for the 8-electrode and 16-electrode EIT sensor. In the case of the 8-electrode sensor, each electrode was 30 mm wide, while in the case of the 16-electrode sensor, each electrode width had to be limited to 15 mm. In order to improve the accuracy of the calculation, the grid was thickened near the electrodes. This model was subsequently used to solve the forward problem and determine the potential distribution on the border of the area. As a consequence, a simulated measurement set was obtained, which was used to attempt the image reconstruction afterwards. For the solution of the inverse problem, the iterative Gauss–Newton algorithm was applied [2,28].

In the second part of the research, particular attention was given to an analysis of the distinguishability of the two separate regions of material with a higher moisture content. For this reason, a model of the sensor, with the shape as described above, was created. Subsequently, two areas with a 30 mm diameter, characterized by higher moisture content, were defined inside the sensor. During several simulations, the distance between those areas was gradually decreased until they became one area in the reconstructed image. The simulation research was carried out for the 8-electrode and the 16-electrode systems. Additionally, the influence of grid density on the quality of imaging the calculation for the grid of increased density was also investigated. The Gauss–Newton algorithm was also used in this part of research.

### 2.3. The EIT Experimental Setup

**Figure 1:**
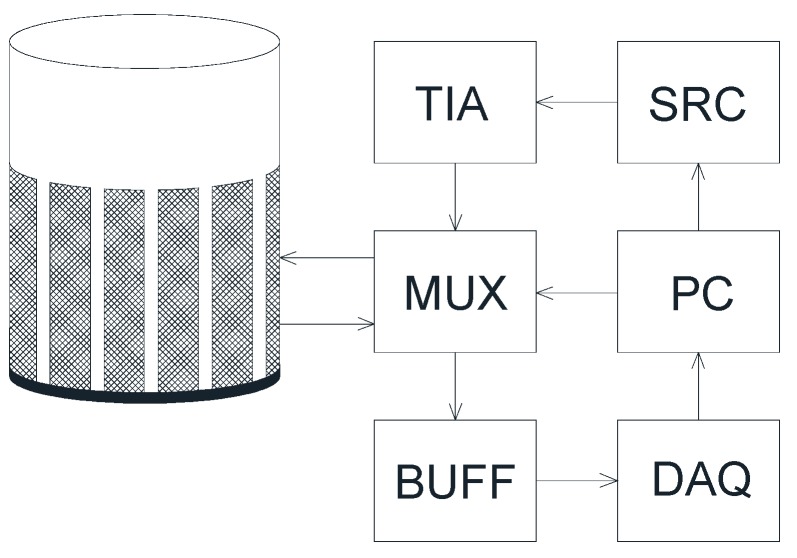
Block diagram of the EIT test stand; TIA: transimpedance amplifier, SRC: voltage source, MUX: switching circuit, PC: computer, BUFF: buffering circuit, DAQ: data acquisition card.

The block diagram of the complete measurement system is shown in the graph (Figure 1). The measurement vessel (the sensor of the tomograph) was constructed using a polycarbonate pipe with an internal diameter of 11 cm and a height of 8 cm. Eight electrodes (sized 7.5 × 3.0 cm), made of copper foil, were circumferentially arranged on the internal side of the vessel. The applied size of the electrodes, unusually large for EIT, results from the need to ensure similar contact impedance between the material and all electrodes. Granular material contacts with the electrodes only on certain points. Providing as many contact points as possible is important, therefore. This results in a drop of contact impedance. Furthermore, an increase of the electrode area in the considered case may contribute to the equalization of the contact impedance on all electrodes.

To allow the sequential switching of the stimulating electrodes, the switching circuit (MUX) was designed and manufactured based on a semiconductor multiplexer and reed relays. This subsystem was controlled by the digital outputs of data acquisition card. In most of the implementations of an impedance tomography, an AC current stimulation (typically several milliamps) is used. This requires the use of a voltage controlled current source (a so-called ‘current pump’), typically the Howland circuit [2,29]. However, a current source is characterized by a significantly higher output impedance than the stimulated load. Due to the very high impedance of the chokeberry (at a low moisture content), it is virtually impossible to build a current source that would allow the generation of stimulus signal in the required frequency range [29,30]. These circumstances caused the need to apply, in this research, voltage stimulation and accurate current measurement systems [2,31]. As the voltage source (SRC), the programmable signal generator Agilent 33500B was used. Since the current flowing through the chokeberry reaches very low values (1–4 µA), it was necessary to develop a current-to-voltage converter (TIA, transimpedance amplifier), characterized by a high amplification factor. For this purpose, the feedback converter circuit, based on operational amplifier OPA2107, itself characterized by the very low bias current, was built [11,29]. This, in turn, allowed the amplification factor to reach 1 V/µA, and the resolution of current measurement was better than 0.2 nA.

The voltage on the electrodes was measured using the high-speed data acquisition card (DAQ), National Instruments PCIe-6351. This is a 16-channel multifunction measurement card with an accurate 16-bit analog-to-digital converter with a maximum sampling rate of 1.25 MHz. The instrument measuring voltage is usually required to have the largest possible input impedance. Although the DAQ card manufacturer reports [32] that the input resistance of the device is very large (>10 GΩ), the significant input capacitance (100 pF) still results in a noteworthy decrease in the input impedance when measuring high frequency voltage. Moreover, the connection cable is characterized by its own capacitance, which is not negligible. In order to reduce the impact of this effect on the achievable measurement accuracy, each input of the DAQ card was equipped with an additional buffering circuit (BUFF) based on the low noise operational amplifier, OPA4228. This allowed for a decrease of approximately 50 times in the input capacitance of the measurement system. The complete stimulus switching and measurement system was controlled using a PC through an application written in LabVIEW [33].

## 3. Results

### 3.1. Impedance Spectrum of the Chokeberry and Its Dependence on Humidity—The Results

**Figure 2:**
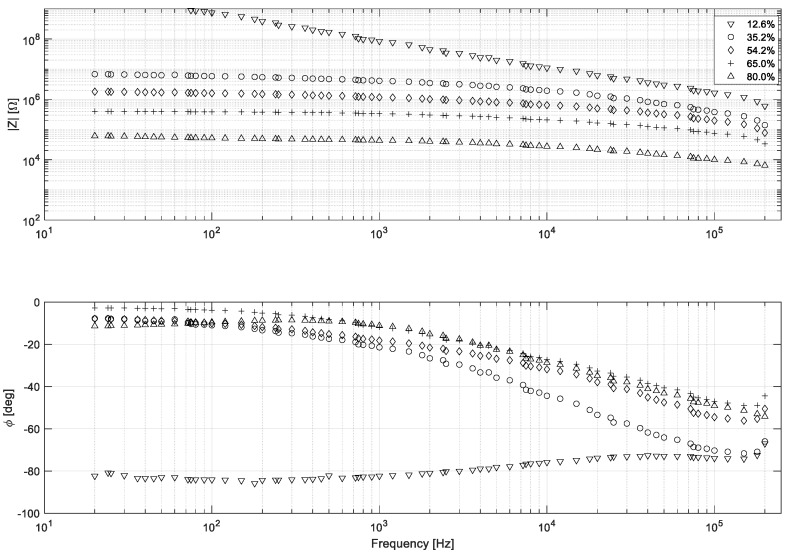
Impedance spectra of the chokeberry, with different moisture contents, at 20 °C.

The results of the experimental research showed that the humidity of the chokeberry is significantly dependent on its electrical properties. As could be expected, absolute impedance explicitly increases by decreasing the moisture content in the chokeberry. This is due to a number of phenomena that occur during the drying of the fruit. The key elements are reducing the number of free ions due to the evaporation of the solvent, as well as reducing the contact surface caused by changes in the structure of the peel [21]. Accordingly, the effect should be present for most of the agricultural crops being dried. It is noteworthy that the results obtained for 12.8% moisture content might be partially charged with higher uncertainty. This is caused by exceeding the LCR bridge measurement range (10^8^ Ω) for frequencies lower than 1 kHz. The obtained results are shown in the graph (Figure 2). 

**Figure 3:**
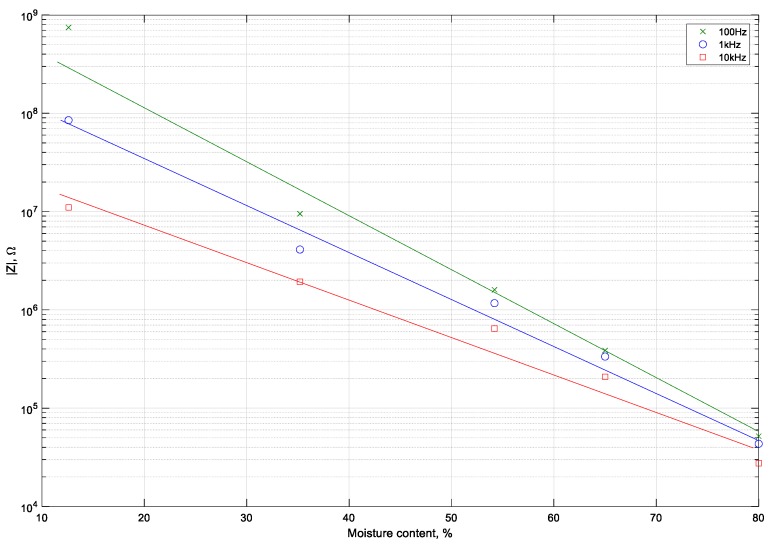
Relationship between the absolute impedance of the chokeberry and its moisture content.

The approximate trends of absolute impedance change with the moisture content in the chokeberry, as visualized in the graph (Figure 3) for selected frequency (100 Hz, 1 kHz, 10 kHz). It could be also stated that the absolute impedance decreases with an increase in frequency. For this reason, utilising a higher frequency makes the measurement easier to conduct, even using low-cost appliances.

The phase angle dependence on humidity of the chokeberry does not show such a clear trend. It is noteworthy that the chokeberry with a low moisture content shows characteristics similar to the dielectric. In this case, particularly at a higher frequency (10^4^–10^5^ Hz), the relationship between the phase angle and the moisture content of the material becomes meaningful. This gives the opportunity to use, at a later stage of the research, both the resistive and capacitive properties of the system by proposing a dual modality tomography [34] method or spectro-tomography [14,15,16]. Further research should obviously include carrying out measurements at different temperatures because, a significant impact on the results might be expected.

During the examination, the phenomenon, which is important in practice, was observed. After the insertion of the chokeberry into the measurement vessel, a gradual decrease in the measured impedance occurred. After approximately 15–50 min (depending on material humidity), the value of the impedance became steady. This phenomenon was stronger the higher the moisture content in the material, and at humidity lower than 30%, the change of impedance was already insignificant. Moreover, the settling time appeared to be influenced by the frequency. For a lower frequency, the settling time reached its minimum, while for higher frequency it increased. The practical importance of this phenomenon should be additionally investigated in future research.

### 3.2. Results of the Simulation Analysis

As shown by the results achieved, described below in detail, identification of the area with the higher humidity is mostly correct. However, even in the case of the simulation data, which is free from noise, material distribution non-uniformity, or inaccuracies in the sensor design, the image reconstruction is not perfect. Outside the area of higher moisture content, the image should be uniform. However, mapping errors (artefacts) can be observed. This indicates a certain limitation of the image reconstruction algorithm.

**Figure 4:**
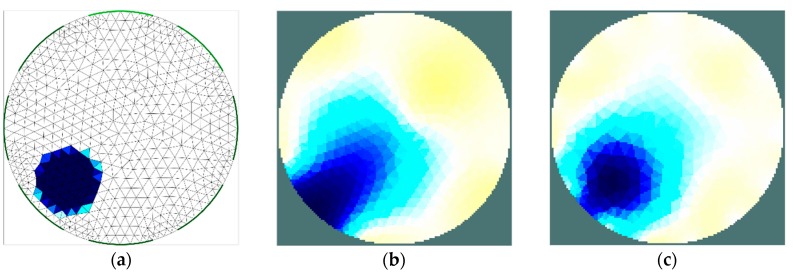
Area of diameter 30 mm located near the border of the sensor—finite element grid (**a**) and image reconstructed using simulated data for the 8-electrode (**b**) and 16-electrode (**c**) sensor.

**Figure 5:**
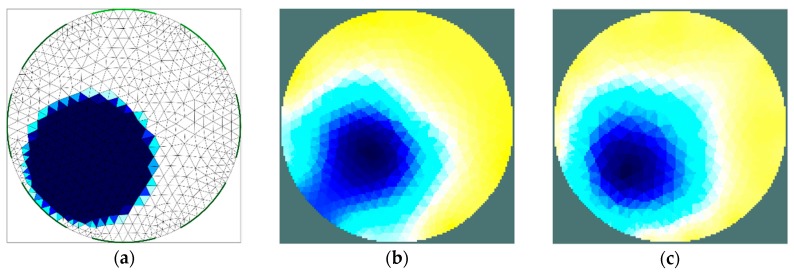
Area of diameter 60 mm located near the border of the sensor—finite element grid (**a**) and image reconstructed using simulated data for the 8-electrode (**b**) and 16-electrode (**c**) sensor.

**Figure 6:**
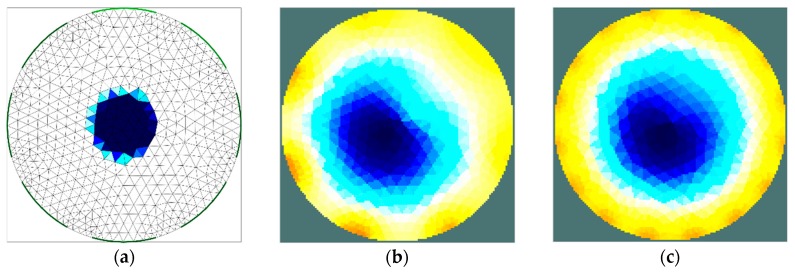
Area of diameter 30 mm located in the centre of the sensor—finite element grid (**a**) and image reconstructed using simulated data for the 8-electrode (**b**) and 16-electrode (**c**) sensor.

**Figure 7:**
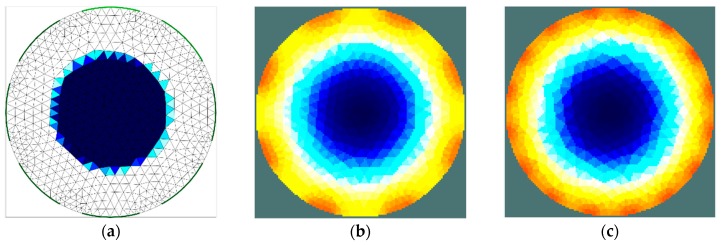
Area of diameter 30 mm located in the centre of the sensor—finite element grid (**a**) and image reconstructed using simulated data for the 8-electrode (**b**) and 16-electrode (**c**) sensor.

The results of the first stage of simulation research (Figure 4, Figure 5, Figure 6 and Figure 7) indicate a good ability to locate a material with higher humidity. Both positions, as well as areas, are obtained precisely enough for the monitoring of the drying processes or control purposes. This confirms the possibility to use the EIT sensor with adopted geometry, as well as the Gauss–Newton algorithm, to achieve the intended purpose. The case where the area of diameter of 30 mm was located in the centre of the sensor (Figure 6) was the exception. In this case, regardless of the sensor used, the designated area was significantly overestimated. Interestingly, in the remaining cases, the results obtained using 8-electrode and 16-electrode sensors did not differ significantly. Obviously, the images obtained with 16-electrode sensor were characterized by a better ability to render the shape of the area. This was particularly noticeable for cases in which the region-of-interest was localized near the border of the sensor (Figure 4 and Figure 5). In the case of area of diameter 60 mm placed in the centre of the sensor (Figure 7), the differences between images acquired using the 8-electrode and 16-electrode system appeared to be negligible.

**Figure 8:**
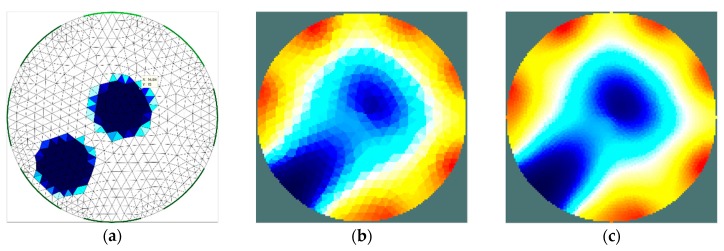
Distinguishability analysis: 8-electrode sensor—finite element grid (**a**) and image reconstructed using simulated data for the normal grid (**b**) and dense grid (**c**).

**Figure 9:**
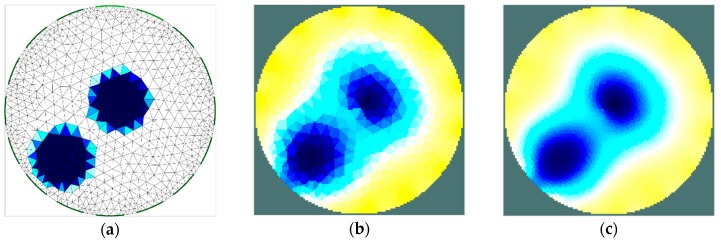
Distinguishability analysis: 16-electrode sensor—finite element grid (**a**) and image reconstructed using simulated data for the normal grid (**b**) and dense grid (**c**).

**Figure 10:**
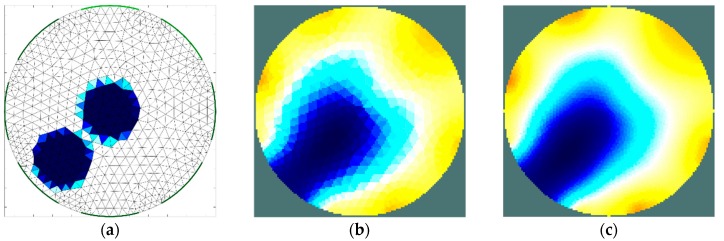
Distinguishability analysis: 8-electrode sensor, regions too close—finite element grid (**a**) and image reconstructed using simulated data for the normal grid (**b**) and dense grid (**c**).

**Figure 11:**
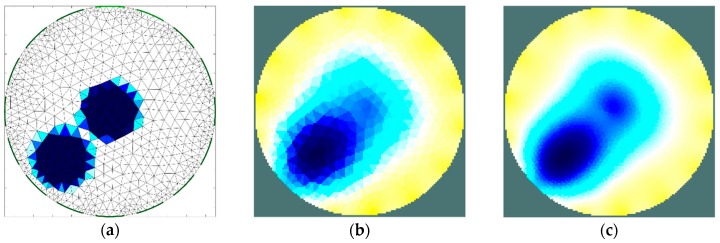
Distinguishability analysis: 16-electrode sensor, regions too close—finite element grid (**a**) and image reconstructed using simulated data for the normal grid (**b**) and dense grid (**c**).

The second stage of simulation research was aimed to estimate the ability of the EIT sensor to identify two separate areas of material with higher moisture content placed inside the sensor. The importance of this feature is related to the relatively low imaging resolution, which is typical of impedance tomography. The results achieved (Figure 8 and Figure 9) illustrate the minimum distance between the areas for which the areas remain disjoint in the reconstructed image. The obtained minimum limit between the borders of the areas (12 mm) was proven to be similar to the radius of these areas. At this stage of the research, the results obtained for the 16-electrode sensor (Figure 8) turned out to be significantly better than the image obtained using the 8-electrode sensor (Figure 7). In each case, the implementation of the dense grid resulted in improved imaging quality. However, the results obtained cannot justify the significant increase in the computation time needed for image reconstruction. In the subsequent graph (Figure 10 and Figure 11), the results of a similar simulation are shown, but in this case, the distance between the borders of the areas was lower than the minimum limit. In this example, there is no possibility to distinguish the areas. However, indentifying the region-of-interest’s existence is possible. To control a drying process, this possibility would also be useful.

### 3.3. Imaging of the Spatial Distribution of Moisture Content in the Chokeberry—Image Reconstruction Based on Experimental Data

Experimental verification of the simulation results was performed using the measurement system described in Section 2.3. The chokeberry was arranged inside the sensor, as in one of the simulation studies (Figure 5). Berries filling the majority of the volume of the sensor had moisture content as follows:

- Case 1: 35.2%;
- Case 2: 54.2%.

However, inside the ‘wet’ area, the moisture of the material was 65.0%. The large difference in humidity between areas in ’case 1’ stems from the need to investigate obtainable imaging contrast. Determining the achievable imaging resolution of the moisture distribution in the material and attempting to improve it will be a further stage of this study.

**Figure 12:**
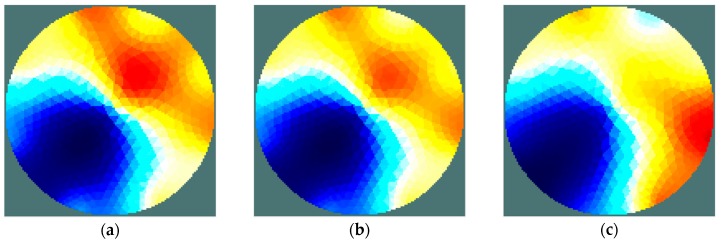
Case 1: Chokeberry with water content of 69.0%/35.2%. Reconstructed images for voltage stimulation of 100 Hz (**a**), 1 kHz (**b**), 10 kHz (**c**).

**Figure 13:**
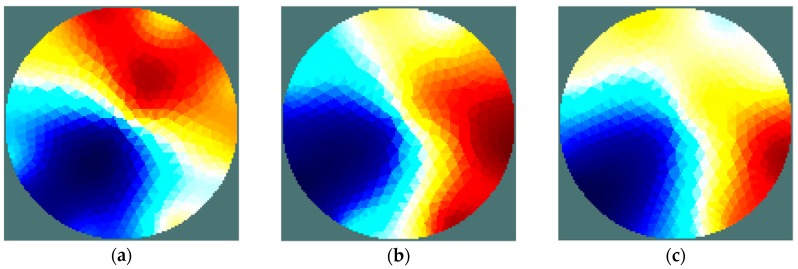
Case 2: Chokeberry with water content of 69.0%/54.2%. Reconstructed images for voltage stimulation of 100 Hz (**a**), 1 kHz (**b**), 10 kHz (**c**).

In the experimental research, a sensing strategy called the ‘opposite electrode pair strategy’ was applied [31]. This strategy means that the voltage source was connected to an opposite pair of electrodes, e.g. 1–5, 2–6, etc. (for the 8-electrode sensor), while the voltage was measured on every electrode. After completion of the voltage measurement, the sourcing electrode pair was switched to the next one. Current drawn from the source was measured simultaneously and maintained at a constant level. Therefore, the complete measurement set is comprised 64 voltage measurements. A measurement was taken for three values of stimulus voltage frequency: 100 Hz, 1 kHz, and 10 kHz. Similar to the simulation study, an iterative Gauss-Newton algorithm was applied to solve the inverse problem. The results of the image reconstruction for each frequency are shown in Figure 12 and Figure 13.

As shown by the results obtained, irrespective of the frequency of the excitation voltage, the location of areas of high humidity can be considered correct. However, it can be seen that all of the reconstructed images are characterized by the artefacts of an intensity significantly greater than in the simulation results. Moreover, the artefacts’ influence on image quality is explicitly dependent on frequency. The quality of imaging clearly increases with a raise in the frequency of the stimulus voltage. It might be a prerequisite to extend the frequency range during future research. In ‘case 2’, the moisture content difference between the areas was lower and led to an increased impact of noise on the measured signals. This, in turn, resulted in an increased presence of artefacts (Figure 13). Presumably, this result also indicates the need to introduce some modifications to the measurement system, especially an increase of the resolution of current measurement. Moreover, a reduction of the noise impact should lead to sensitivity improvement for whole EIT system. 

In this stage of the study, the experimental research was focused mainly on proving the feasibility of humidity imaging with the applied method. For this reason, only one material arrangement was tested. Any quantitative indicators of the quality of imaging were not introduced at this stage of the research. Undoubtedly, in further research, such indicators should be introduced, giving a more detailed comparison of the results. Since the method needs to be additionally validated, in the next season of fruit harvest, the research will be continued. Further research should also be concerned with improving measurement accuracy, as well as imaging resolution. Additional humidity ranges, different positions, and sizes of the region of interest and multiple-region detection have been shown to be particularly important for further investigation.

## 4. Discussion

The study showed a significant correlation between the absolute impedance of the chokeberry and its moisture content in a wide frequency range. This indicates the possibility to use the impedance measurement to assess moisture content. In practice, the condition for the correct interpretation of the interdependence of impedance and humidity should take into account temperature compensation—in this study, it was not analysed. Attention was drawn to the phenomenon of long-term stabilization of the measurement result after insertion of the chokeberry into the sensor. That phenomenon limits, but does not exclude, the application of the presented method to dryers characterized by periodic mixing of the material. The high impedance of the chokeberry requires an unusual approach to design the measurement system of the EIT. It was proven that the application of a voltage stimulation and stimulus current measurement, together with additional buffering of the data acquisition card, made the EIT imaging feasible.

An important issue is ensuring good contact between the tested material and the electrodes. The fact that granulated materials contact with the electrodes only on a finite number of points may lead to a difference between the contact impedance on each electrode. For this reason, it is useful to use electrodes with a relatively large area. However, increasing the electrodes’ area limits the number of electrodes able to be installed inside the sensor, which in turn limits its imaging resolution. On the basis of the simulation results, the use of an 8-electrode sensor instead of a 16-electrode sensor might be justified in the considered EIT system.

Identification of the insufficiently dried material can be regarded as satisfactory despite the above-mentioned issues. However, the artefacts appearing in the images make interpretation of the results difficult. However, these artefacts might be significantly reduced through minor modifications of the measurement system. This was shown, in particular, by the comparison between the simulation and experimental results. Since the quality of imaging clearly increases by raising the frequency of the stimulus voltage, future research should be focused on a wider frequency range.

The accuracy achievable with other image reconstruction algorithms has not been studied in this paper, but the results acquired using impedance spectroscopy suggest that it will be possible to improve the imaging quality by taking into account both the real and imaginary parts of complex impedance. Especially at a low moisture content in the material being dried, the measurement of the phase angle seems to be purposeful. Nevertheless, the obtained results indicate the prospective usefulness of the presented methods in the practical, non-intrusive monitoring and control of drying processes.
